# Comparison between effectiveness of Mechanical and Manual Traction combined with mobilization and exercise therapy in Patients with Cervical Radiculopathy

**DOI:** 10.12669/pjms.321.8923

**Published:** 2016

**Authors:** Syed Rehan Iftikhar Bukhari, Syed Shakil-ur-Rehman, Shakeel Ahmad, Aamer Naeem

**Affiliations:** 1 Dr. Syed Rehan Iftikhar Bukhari, MS-OMPT, Lecturer, College of Physical Therapy, Government College University Faisalabad, Pakistan; 2 Dr. Syed Shakil-ur-Rehamn, MS(MSKPT), Principal/ Associate Professor, Riphah College of Rehabilitation Sciences, Riphah International University, Islamabad, Pakistan; 3 Dr. Shakeel Ahmad, MS(NMSKPT), Assistant Professor, Riphah College of Rehabilitation Sciences, Riphah International University, Islamabad, Pakistan; 4 Dr. Aamer Naeem, Post Professional DPT, Lecturer, Riphah College of Rehabilitation Sciences, Riphah International University, Islamabad, Pakistan

**Keywords:** Cervical Radiculopathy, Mechanical Traction, Manual Traction, Segmental Mobilization, Exercise Therapy

## Abstract

**Background and Objective:**

Cervical radiculopathy is a common neuro-musculo-skeletal disorder causing pain and disability. Traction is part of the evidence based manual physical therapy management due to its mechanical nature, type of traction and parameters related to its applicability and are still to be explored more through research. Our objective was to determine the Effects of Mechanical versus Manual Traction in Manual Physical Therapy combined with segmental mobilization and exercise therapy in the physical therapy management of Patients with Cervical Radiculopathy.

**Methods:**

This randomized control trial was conducted at department of physical therapy and rehabilitation, Rathore Hospital Faisalabad, from February to July 2015. Inclusion criteria were both male and female patients with evident symptoms of cervical spine radiculopathy and age ranged between 20-70 years. The exclusion criteria were Patients with history of trauma, neck pain without radiculopathy, aged less than 20 and more than 70. A total of 72 patients with cervical radiculopathy were screened out as per the inclusion criteria, 42 patients were randomly selected and placed into two groups by toss and trial method, and only 36 patients completed the study, while 6 dropped out. The mechanical traction was applied in group A and manual traction in group B along with common intervention of segmental mobilization and exercise therapy in both groups for 6 weeks. The patient’s outcomes were assessed by self reported NPRS and NDI at the baseline and after completion of 06 weeks exercise program at 3 days per week. The data was analyzed through SPSS version-21, and paired T test was applied at 95% level significance to determine the statistical deference between two groups.

**Results:**

Clinically the group of patients treated with mechanical traction managed pain (mean pre 6.26, mean post 1.43), and disability (mean pre 24.43 and mean post 7.26) more effectively as compared with the group of patients treated with manual traction (Pain mean pre 6.80, mean post 3.85 and disability mean pre 21.92 and post 12.19). Statistically the results of both mechanical and manual traction techniques are equally significant in group A and B for pain and disability (p-value less than 0.05).

**Conclusion:**

If patients of cervical radiculopathy treated with mechanical traction, segmental mobilization, and exercise therapy will manage pain and disability more effectively than treated with manual traction, segmental mobilization, and exercise therapy.

## INTRODUCTION

Cervical radiculopathy is a common neuro-musculo-skeletal disorder causing pain and disability. Pain perceived as arising in the arm caused by the irritation of cervical spinal nerves or its roots is considered as radicular pain and termed as radiculopathy, and 1 out of 1000 individuals suffer from cervical radiculopathy.^1^ Radicular pain is commonly managed by pharmacologic management, physical therapy and rehabilitation measures, interventional techniques and surgical treatments. The gold slandered is that conservative management is more effective than surgical management.^2^

Manual Physical Therapy is a part of conservative management and effective in managing pain, joint restrictions and disability, while applied in combination with therapeutic exercises.^3^ the Cervical traction combined with exercise therapy has the additional effects in reducing pain, and function, while managing neck pain conservatively by manual physical therapy.^4^ The soft cervical collar, rest for three to six weeks, physiotherapy accompanied by home exercises for six weeks is more beneficial in managing acute radicular pain as compared to wait and see strategy.^5^

It is also evident that Manual traction techniques combined with cervical mobilization improves pain and manages disability in patients with cervical radiculopathy as compared with mobilization alone.^6^ Literature supports the effectiveness of traction techniques, but the effectiveness of different types of traction and parameters related to its applicability is still to be more elaborated by research. The current study was designed to determine the Effects of Mechanical versus Manual Traction combined with segmental mobilization and exercise therapy in of Patients with Cervical Radiculopathy.

## METHODS

This randomized control trial was conducted at department of physical therapy and rehabilitation, Rathore Hospital Faisalabad, from February to July 2015. Inclusion criteria were both male and female patients with cervical spine involvement, evident radicular symptoms, and age ranged between 20-70 years. Patients with history of trauma, neck pain without radiculopathy, aged less than 20 and more than 70 were excluded. The total of 72 patients with cervical radiculopathy were screened out as per the inclusion criteria, 42 were randomly selected and placed into two groups by toss and trial method, and only 36 patients completed the study, while 6 dropped out. Out of 36 patients, 24 (66%) were male and 12 (34%) female, and age ranged 21-62 years and mean age 45.78 years.

Mechanical and manual are two commonly used types of tractions techniques were applied in two different groups of patients in combination with segmental mobilization and exercise therapy. The outcomes were assessed by numeric Pain rating scale (NPRS) for pain and neck disability index (NDI) for function. NPRS is a valid and reliable tool for the assessment of pain in patients with neuro-musculo-skeletal problems.^7^,^8^ NDI is another valid and reliable tool and has sufficient support for assessing function accurately in patients with mechanical neck disorders.^9^,^10^

The mechanical traction was applied in group A and manual traction in group B along with common intervention of segmental mobilization and exercise therapy in both groups for 6 weeks. Mechanical traction was applied in supine position by manually adjusted mechanical traction equipment, with 10 second pull and 5 second rest for 10 minutes in single session in group A. The traction force was equal to 10-15% of body weight of each patient and calculated prior to intervention. Manual traction was applied in supine position at 25 degree neck flexion with 10 second pull and 5 second rest for 10 times in single session in group B. Followed C-3 to C-7 segments was mobilized by central posterior anterior (CPA) glide in prone position at and each glide was sustained by 5 seconds for 10 reputations per session in both groups. Active Range of motion, stretching and isometric strengthening home exercise program was advised to all patients in both groups. The outcomes of the study were assessed by NRS and NDI at the baseline, and after completion of 06 weeks exercise program at 3 days per week. The data was analyzed through SPSS version-21, and paired T test was applied at 95% level significance to determine the statistical deference between two groups.

## RESULTS

Clinically the group of patients treated with mechanical traction manage pain (mean pre 6.26, mean post 1.43), and disability (mean pre 24.43 and mean post 7.26) more effectively as compared with the group of patients treated with manual traction. (Pain mean pre 6.80, mean post 3.85 and disability mean pre 21.92 and post 12.19). Whereas statistically the results of both mechanical and manual traction techniques are equally significant in group A and B for pain and disability (p-value less than 0.05) Table-I.

**Table-I:** Clinical and statistical comparison of group A and B.

Study variables	Statistical parameters	Group A : Treated with Mechanical traction, segmental mobilization and Exercise therapy (n=15)		Group B : Treated with Manual traction, segmental mobilization and Exercise therapy (n=21)	

		Pre	Post	Pre	Post
PainonNPRS	Mean	6.26	1.43	6.80	3.85
	SD	1.20	1.04	1.20	1.68
	p-value	0.000	0.000		
FunctiononNDI	Mean	24.43	7.20	21.92	12.19
	SD	8.64	4.42	8.89	6.74
	p-value	0.000	0.000		

The results as shown in Table-I were more effective for the group of patients treated with mechanical traction, segmental mobilization, and exercise therapy in managing pain, and disability as compared to the group of patients treated with manual traction, segmental mobilization, and exercise therapy as measured by NPRS and NDI.

## DISCUSSION

The result of the current study demonstrated that both pain and function were improved in both groups but the group of patients treated with mechanical traction managed pain (mean pre 6.26, mean post 1.43), and disability (mean pre 24.43 and mean post 7.26) more effectively as compared with the group of patients treated with manual traction (Pain mean pre 6.80, mean post 3.85 and disability mean pre 21.92 and post 12.19), as the outcomes were measured by NPRS and NDI. Statistical results of both the groups were significant (p-values less than 0.05), while clinically the mechanical traction shows added improvement in pain and function. The reason is probably the traction force managed by the traction machine is uniform throughout the session while the traction force applied manually is difficult to keep it uniform due fluctuations in muscle activity, which is natural.

Langevin and colleagues conducted a randomized control trial on compared the 2 Manual Therapy and Exercise Protocols for Cervical Radiculopathy. The objective was to compare a rehabilitation program with the idea to increase the size of the intervertebral foramen (IVF) of the involved nerve root to a rehabilitation program that doesn’t include any specific techniques thought to increase the size of the IVF in patients presenting with cervical radiculopathy. The outcomes were assessed by NDI, shortened version of the Disabilities of the Arm, Shoulder and Hand questionnaire, NPRS evaluated at baseline, at the end of the 4-week program (week 4), and 4 weeks later (week 8). They concluded that manual therapy techniques and therapeutic exercises are effective in reducing pain and functional limitations caused by cervical radiculopathy.^11^

Fritz and colleagues carried out a randomized control trial with the title exercise only, exercise combined with mechanical traction, or exercise with over-door traction for patients with cervical radiculopathy, with or Without Consideration of Status on a Previously Described Sub grouping Rule. The objective was to investigate the effectiveness of cervical traction along with exercise for targeted subgroups of patients with neck pain. The outcomes were assessed by NDI at baseline 3 months, 6 months, and 12 months. They concluded combination mechanical traction and exercise for patients with cervical radiculopathy enhance function and reduce pain.^4^

Cleland and group conducted a case series on 11 patients and the objective was to examine the outcomes of a consecutive series of patients coming to physical therapy with cervical radiculopathy and managed with the use of manual physical therapy techniques, cervical traction, and strengthening exercises program. The outcomes were measured by NPRS, NDI, Patient-Specific Functional Scale (PSFS), and global rating of change (GROC) at the time of discharge from therapy and at a 6-month follow-up session. Ten out of 11 (91%) demonstrated positive change in pain and function.^12^

## CONCLUSION

It is concluded that if patients of cervical radiculopathy treated with mechanical traction, segmental mobilization, and exercise therapy will manage pain and disability more effectively than treated with manual traction, segmental mobilization, and exercise therapy. Furthermore a study with large sample size and prolonged duration of intervention is recommended.
